# Network Pharmacology Combined with Molecular Docking and Experimental Verification Reveals the Bioactive Components and Potential Targets of Danlong Dingchuan Decoction against Asthma

**DOI:** 10.1155/2022/7895271

**Published:** 2022-02-10

**Authors:** Beibei Xue, Qingyang Zhao, Di Chen, Xia Wang, Lanxuan Li, Jianbao Li, Jinna Tian

**Affiliations:** ^1^Clinical Medical College, Chengdu University of Traditional Chinese Medicine, Chengdu, 610075, China; ^2^Chengdu Xinjin District Hospital of Traditional Chinese Medicine, Chengdu, 611430, China; ^3^Suining First People's Hospital, Suining, 629000, China; ^4^Zigong Hospital of Traditional Chinese Medicine, Zigong 643000, China; ^5^Affiliated Hospital of Chengdu University of Traditional Chinese Medicine, Chengdu 610072, China

## Abstract

**Background:**

Danlong Dingchuan Decoction has a definite effect in the clinical treatment of asthma. This study aimed to explore the material and molecular biological basis of Danlong Dingchuan Decoction in treating asthma through network pharmacology combined with animal experiments.

**Materials and Methods:**

First, the chemical constituents of Danlong Dingchuan Decoction were screened from the Traditional Chinese Medicine Systematic Pharmacology Analysis Platform (TCMSP) and the Traditional Chinese Medicine and Chemical Composition Database. Literature reports on asthma targets were obtained from the Online Mendelian Inheritance in Man (OMIM), Therapeutic Targets Database (TTD), and other databases. Then, the protein-protein interaction network was constructed according to the matching results of Danlong Dingchuan Decoction and asthma targets. Furthermore, Gene Ontology (GO) and Kyoto Encyclopedia of Genes and Genomes (KEGG) analyses were performed by the Database for Annotation, Visualization, and Integrated Discovery (DAVID). Finally, the interaction between the active compounds of Danlong Dingchuan Decoction and key targets was simulated using molecular docking. In animal experiments, ovalbumin was used to induce asthma in mice. After treating the mice by oral gavage administration of Danlong Dingchuan Decoction, the expression levels of tumor necrosis factor-*α* (TNF-*α*) and interleukin-1*β* (IL-1*β*) were detected in the lung tissue of the mice by enzyme-linked immunosorbent assay kit, whereas TLR4 mRNA expression was detected by quantitative reverse transcription-polymerase chain reaction.

**Results:**

A total of 247 active compounds and 155 potential targets were obtained. Enrichment analysis showed that quercetin, xanthine, lysine, kaempferol, *ß*-sitosterol, and four other active compounds were the main components of Danlong Dingchuan Decoction; IL-6, TNF, CXCL8, VEGFA, MAPK3, IL-10, PTGS2, IL-1*β*, IL-4, and TLR4 were the potential targets for therapy. KEGG analysis showed that the cAMP signaling pathway, cGMP-PKG signaling pathway, NF-*κ*B signaling pathway, and PI3K-Akt signaling pathway might play an important role in treating asthma. Molecular docking analysis showed that quercetin combined well with TNF, CXCL8, and TLR4. Animal experiments showed that Danlong Dingchuan Decoction effectively reduced the expression levels of TNF-*α*, IL-4, TGF-*β*1, IL-6, IL-8, and IL-1*β* in the lung tissue of asthmatic mice and inhibited TLR4 mRNA expression.

**Conclusions:**

Danlong Dingchuan Decoction may act on key targets (such as IL-6, TNF, CXCL8, VEGFA, and MAPK3) with key active ingredients (such as quercetin, xanthine, lysine, kaempferol, and *ß*-sitosterol) to reduce the expression levels of IL-4, IL-6, IL-8, and other Th2 cytokines. This may be the mechanism by which Danlong Dingchuan Decoction reduces airway inflammation and treats asthma mediated by Th2 cytokines.

## 1. Introduction

Asthma is a heterogeneous chronic inflammatory disease of the lower respiratory tract that is influenced by a variety of cells and cellular components. It is the most common chronic respiratory disease in children, and its main clinical signs include recurrent attacks of wheezing, cough, chest distress, and shortness of breath. The pathogenesis of asthma is complex. Modern immunological studies have shown that excessive Th2 response leading to enhanced recruitment of eosinophils and immunoglobulin *E* (IgE) mediated inflammation is the main driver of asthma [1]. There are almost 300 million patients with asthma worldwide, and more than 200,000 people die of asthma each year. It is estimated that there will be 400 million patients with asthma worldwide by 2025 [2, 3]. Chronic and recurrent asthma attacks not only can lead to airway remodeling, lung function injury, and irreversible airway obstruction but can also seriously deteriorate the physical and mental health of patients and reduce their quality of life. In recent years, the incidence of asthma in children and the corresponding mortality rates have been increasing. Therefore, the prevention and treatment of bronchial asthma are a worldwide public health problem.

In recent years, the comprehension and research of asthma in Traditional Chinese Medicine (TCM) have become increasingly mature. Clinical trials and animal experiments have confirmed that TCM compounds or extracts are effective in asthma prevention and treatment, suggesting that the overall regulation of TCM with multitargets and multichannels has potential advantages in treating asthma. This shows a good prospect to explore an effective antiasthma TCM or compound decoction under the guidance of TCM basic theory.

Danlong Dingchuan Decoction is a clinically effective decoction for treating infantile asthma. It originates from Jinshui Liujun Decoction in “Jingyue Quanshu” and is composed of Danshen, Dilong, Danggui, Shudihuang, Fabanxia, Chenpi, Fuling, Shegan, Kuandonghua, Baishao, and Gancao. The whole decoction treats asthma by making reinforcement and elimination in combination and has the effects of “tonifying kidney and penetrating evil qi.” Our preliminary clinical and experimental studies have found no significant difference in the effective clinical rate between Danlong Dingchuan Pill and fluticasone aerosol in treating children with bronchial asthma in the remission stage. Eosinophils and IgE significantly decreased, and pulmonary function significantly improved after treatment. Danlong Dingchuan Decoction improved the airway remodeling process of asthmatic mice, inhibited interleukin-4 (IL-4) overexpression in the lung tissue, transformed growth factor-*β*1 (TGF-*β*1) in the serum, promoted interferon-*γ* (IFN-*γ*) secretion, reduced HMGB1 and *α*-smooth muscle actin (*α*-SMA) contents in the lung tissue, and reduced MMP9 and TIMP1 in the serum. These results suggest that Danlong Dingchuan Decoction has a multitarget and multilevel overall regulatory effect on the complex immune-inflammatory microenvironment and airway remodeling pathological process of asthma [4–6]. However, its specific pharmacological effects still need to be further explored.

Network pharmacology integrates chemical informatics, bioinformatics, biological networks, network analysis, traditional pharmacology, and other disciplines' knowledge. It is based on computer-aided analysis that combines TCM components, disease targets, and biological signaling pathways. Considering the interaction between drugs from the overall point of view, network pharmacology is in line with the complexity and diversity of TCM decoction. Therefore, network pharmacology has widely been used in the study of TCM compounds in recent years [7].

Based on the network pharmacology method, this study investigated the mechanism of Danlong Dingchuan Decoction in treating asthma. Combined with animal experiments to verify the predicted results, this study provided a relevant basis for subsequent research.

## 2. Materials and Methods

### 2.1. Network Pharmacology

#### 2.1.1. Thorough Investigation of the Active Ingredients and Targets of Danlong Dingchuan Decoction

Based on the Pharmacopoeia of the People's Republic of China (2020 Edition) [8], the standardized names of the 11 herbs in Danlong Dingchuan Decoction are as follows: Danshen (Salviae Miltiorrhizae Radix et Rhizoma), Dilong (Pheretima), Danggui (Angelicae Sinensis Radix), Shudihuang (Rehmanniae Radix Praeparata), Fabanxia (Pinelliae Rhizoma Praeparatum), Chenpi (Citri Reticulatae Pericarpium), Fuling (Poria), Shegan (Belamcandae Rhizoma), Kuandonghua (Farfarae Flos), Baishao (Paeoniae Radix Alba), and Gancao (Glycyrrhizae Radix et Rhizoma). The chemical constituents of the 11 herbs in Danlong Dingchuan Decoction were searched in Traditional Chinese Medicine Systematic Pharmacology Analysis Platform (TCMSP) (https://tcmspw.com/tcmsp.php), TCM and chemical composition database (http://www.organchem.csdb.cn), and literature reports. “ADME” refers to the progress of Absorption, Distribution, Metabolism, and Excretion of exogenous chemicals by the body. The qualified active compounds and their targets were screened according to the two ADME attribute values of oral bioavailability (OB) ≥ 30% and drug-likeness (DL) ≥ 0.18.

#### 2.1.2. Collection and Pretreatment of Asthma Target Proteins

GeneCards (https://www.genecards.org/), Online Mendelian Inheritance in Man (OMIM) (https://omim.org/), Therapeutic Targets Database (TTD) (http://db.idrblab.net/ttd/) databases, and DrugBank (https://www.drugbank.ca/) database were searched using “asthma” as the keyword to collect asthma-related targets. The contents of multiple databases were merged, and duplicate values were removed to obtain the disease targets of asthma. To standardize the protein target information, the species was limited to “*Homo sapiens*.” The protein target information of “Reviewed (Swiss-Prot)” was authenticated in the UniProt database (https://www.uniprot.org/), and the protein targets were transformed into the corresponding gene names.

#### 2.1.3. Active Ingredients of Danlong Dingchuan Decoction: Construction of the Asthma Target Protein-Protein Interaction (PPI) Network

Using Venny 2.1 online tool (http://bioinfogp.cnb.csic.es/tools/venny/index.html.), the obtained asthma disease targets were matched with the active compound targets of Danlong Dingchuan Decoction and the common genes were obtained, which were the potential targets of Danlong Dingchuan Decoction in treating asthma. The active compounds in Danlong Dingchuan Decoction that fail to act on asthma were eliminated. Next, the remaining compounds and common targets were imported into the String database (https://string-db.org/) to construct the target PPI network of Danlong Dingchuan Decoction on asthma. The “TCM-active compounds-disease targets” network diagram was constructed by Cytoscape 3.7.1 [9], and the network was analyzed and visualized.

#### 2.1.4. Gene Ontology (GO) and Kyoto Encyclopedia of Genes and Genomes (KEGG) Enrichment Analyses

Gene Ontology (GO) and Kyoto Encyclopedia of Genes and Genomes (KEGG) analyses were performed on the targets of Danlong Dingchuan Decoction on asthma through the Database for Annotation, Visualization, and Integrated Discovery (DAVID) database (https://david.ncifcrf.gov/summary.jsp), with *P* < 0.05 as the criterion. The GO analysis selected the top 10 enrichment results with the lowest *P*-value, while the KEGG analysis selected the top 20 signaling pathways with the lowest *P*-value for analysis. The data processing platform (http://www.bioinformatics.com.cn) was used to visualize the results.

#### 2.1.5. Main Active Ingredients of Danlong Dingchuan Decoction: Targets of Molecular Docking

The potential targets of Danlong Dingchuan Decoction on asthma were docked with the main compounds of Danlong Dingchuan Decoction by AutoDockTools 1.5.6 [10]. Greater stability of the ligand and receptor binding conformation indicated a higher probability that the effect would occur.

### 2.2. Experimental Verification

#### 2.2.1. Animals

The experiments described in this study were approved by the Animal Ethics Committee of Chengdu Medical College in accordance with the guide for “Animal Research: Reporting of In Vivo Experiments (ARRIVE)” with the approval number “CDMCDL2018009.” A total of 180 healthy male balb/*c* mice of Specific Pathogen Free (SPF) grade weighing 18–20 g were purchased from Chengdu Dashuo Experimental Animal Co., Ltd. (Chengdu, China) with the animal license number “SCXK2015-030.” All of the mice were reared in cages at the Animal Laboratory Center of Chengdu University of Traditional Chinese Medicine; they were housed in standard breeding cages at 22°C with a 12/12-h light/dark cycle (light on from 07 : 00 to 19 : 00) and had free access to food and water. All possible efforts were made to minimize animal suffering and to reduce the number of animals used per condition by calculating the necessary sample size before performing the experiments, and all of the animals were randomly assigned to cohorts when used. When the experiment was performed, all of the animals were euthanized.

#### 2.2.2. Preparation of Experimental Drugs

Danlong Dingchuan Decoction is composed of Danshen 10 g (B N.: 161201), Dilong 10 g (B N.: 170101), Danggui 10 g (B N.: 160901), Shudihuang 10 g (B N.: 1604031), Fabanxia 10 g (B N.: 161102), Chenpi 10 g (B N.: 171001), Fuling 10 g (B N.: 161201), Shegan 10 g (B N.: 170201), Kuandonghua 10 g (B N.: 161210), Baishao 10 g (B N.: 170301), and Gancao 6 g (B N.: 161001). All these herbs were provided by the Pharmacy Department of the Affiliated Hospital of the Chengdu University of TCM and were identified as authentic. All of the herbs were soaked in pure water for 2 h, decocted for 20 min, and subsequently filtered and stored. The filter residue was decocted with pure water for 15 min, filtered, and mixed with the liquid decocted for the first time. After decoction two times, a slow fire was concentrated until the Danlong Dingchuan Decoction dose was 1 g mL^−1^. The decoction was stored in a refrigerator at 4°C. Prednisone acetate tablet was prepared into 0.42 g L^−1^ suspension (5 mg/tablet, B N.: 388001), Chongqing Kerui Pharmaceutical Group Co., Ltd. (Chongqing, China).

#### 2.2.3. Grouping, Modeling, and Dosing

To reflect the therapeutic effects of Danlong Dingchuan Decoction more objectively, prednisone acetate, a common clinical drug, was used as a comparison. A total of 180 mice were randomly divided into a blank control group, a model group, a prednisone group, a high-dose Danlong Dingchuan Decoction group (twice the clinical equivalent dose), a medium-dose Danlong Dingchuan Decoction group (equivalent to the clinical equivalent dose), and a low-dose Danlong Dingchuan Decoction group (equivalent to half of the clinical equivalent dose), with 30 mice in each group. Except for the blank control group, the mice in other groups were sensitized by an intraperitoneal injection of 0.2 mL antigen liquid (containing 100 *μ*g ovalbumin (OVA) and 1 mg aluminum hydroxide dry powder) on day 1 and day 14 of the experiment. From day 28, 1% OVA solution was atomized and inhaled for 5 consecutive days, 30 min each time, once daily. From day 35, aerosol inhalation was started to trigger asthma thrice weekly for 6 weeks. In the blank control group, physiological saline was used instead. From day 35, oral gavage administration was started simultaneously. Before daily atomization stimulation, both the blank control and the model groups were administered 0.9% physiological saline (10 mL kg^−1^ day^−1^) and prednisone acetate (13.86 mg kg^−1^ day^−1^). According to Shi Xinyou's “Modern Medical Experimental Zoology,” the dosages of the high-, medium-, and low-dose Danlong Dingchuan Decoction groups were converted according to the human and animal body surface area ratio. The dosage of a mouse (g/kg) equals the dosage of a human ((g)/60 kg × 9.1). Therefore, high-dose Danlong Dingchuan Decoction was 39.84 g kg^−1^ day^−1^, medium-dose Danlong Dingchuan Decoction was 26.56 g kg^−1^ day^−1^, and low-dose Danlong Dingchuan Decoction was 13.28 g kg^−1^ day^−1^. All drugs were administered for 42 consecutive days.

#### 2.2.4. Effects of Danlong Dingchuan Decoction on the Pathological Changes of Lung Tissue in Asthmatic Mice

Forty-two days after drug intervention, six mice were randomly selected from each group. Lung tissue was separated after cervical dislocation, and the upper and middle lobes of the right lung were fixed in 4% paraformaldehyde treated with diethylpyrocarbonate (DEPC) water. After conventional paraffin embedding, they were sliced (4 *μ*m thick), and hematoxylin and eosin (H&E) staining was performed to observe the pathological changes of lung tissue under a microscope. H&E staining was used to evaluate airway inflammation in each group, and the scoring standard was set in accordance with the literature [11] as follows: 0 points = no inflammatory cells around the airway; 1 point = a few inflammatory cells around the airway; 2 points = many inflammatory cells with uneven distribution around the airway, or the inflammatory cells form a ring with a thickness of one cell; 3 points = a large number of uniformly distributed inflammatory cells around the airway, rarely clumping or forming a ring of inflammatory cells; and 4 points = a large number of inflammatory cells clustered around the airway, or the inflammatory cells form a ring with a layer thickness of more than four cells.

#### 2.2.5. Determination of the Expression Levels of TNF-*α*, IL-4, TGF-*β*1, IL-6, IL-8, and IL-1*β* in Lung Tissues of Asthmatic Mice by ELISA

Forty-two days after drug intervention, 10 mice from each group were randomly selected for immunohistochemical detection in line with the double-antibody Sandwich ABC-ELISA method. Img-Pro6.0 image analysis system was used to observe each section at ×200 magnification. Positive expression was brownish yellow after staining. TNF-*α*, IL-4, TGF-*β*1, IL-6, IL-8, and IL-1*β* expression levels in the lung tissue were calculated in each of the groups.

#### 2.2.6. Effects of Danlong Dingchuan Decoction on TLR4 mRNA Expression in the Lung Tissue of Asthmatic Mice with Reverse Transcription-Polymerase Chain Reaction (RT-PCR)

Forty-two days after drug intervention, six mice were randomly selected from each group. Total RNA was extracted from the lung tissue in each group, and cDNA was synthesized in accordance with the TLR4 kit instructions for quantitative RT-PCR. Primer sequences used are presented in Table 1.

### 2.3. Statistical Analysis

SPSS 25.0 software (IBM Corp., Armonk, NY, USA) was used for statistical analysis, and the measurement data were expressed as mean ± standard deviation (x̄ ± *s*). One-way analysis of variance (ANOVA) was used for comparison between the groups, and the SNK-q test was used for pairwise comparison. GraphPad Prism 8.0 (GraphPad Software, Inc., San Diego, California, USA) for windows was used to draw the statistical graphs. *P* < 0.05 was considered statistically significant.

## 3. Results

### 3.1. Results of Network Pharmacology

#### 3.1.1. Active Ingredients of Danlong Dingchuan Decoction

The chemical constituents of 11 herbs contained in Danlong Dingchuan Decoction were searched in the TCMSP database. Under the conditions of OB ≥ 30% and DL ≥ 0.18, a total of 247 active compounds were obtained and passed the repetition, including 59 compounds from Danshen, 38 compounds from Dilong, 2 compounds from Danggui, 2 compounds from Shudihuang, 12 compounds from Fabanxia, 5 compounds from Chenpi, 6 compounds from Fuling, 11 compounds from Shegan, 16 compounds from Kuandonghua, 8 compounds from Baishao, and 88 compounds from Gancao, including quercetin, kaempferol, *ß*-sitosterol, xanthine, and others (Supplementary Materials, Table S1). In all, 550 gene targets were obtained after the removal of duplicate targets in 247 active compounds (Supplementary Materials, Table S2).

#### 3.1.2. Intersection Targets of Danlong Dingchuan Decoction and Asthma

**Figure 1:**
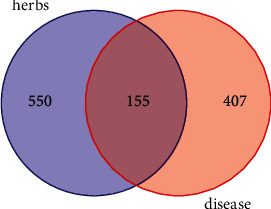
The overlapping targets of Danlong Dingchuan Decoction and asthma with Venny. The red circle indicates the targets of asthma, the purple circle indicates the targets of Danlong Dingchuan Decoction, and the intersecting part of the two circles indicates the common targets of both.

Combined with GeneCards, OMIM, TTD, and other databases, 407 asthma-related targets were obtained. A total of 155 overlapping targets between Danlong Dingchuan Decoction and asthma are shown in a Venn diagram (Figure 1). The overlapping targets were the potential targets for Danlong Dingchuan Decoction in treating asthma.

#### 3.1.3. Herb-Compound-Target-Disease Network Analysis

**Figure 2:**
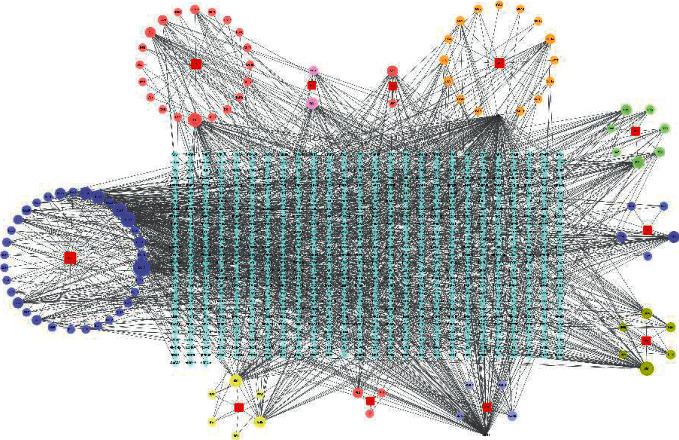
Active compounds-targets diagram of Danlong Dingchuan Decoction. The blue square indicates the intersection target of disease and compounds, the red square indicates the names of the herbs, and circles indicate compounds.

Cytoscape 3.7.1 was used to connect the compounds with the targets, and the network diagram of “herb-compound-target” was obtained (Figure 2). The network showed 821 nodes, including one disease, 11 herbs, 247 compounds, and 155 potential targets. Among them, the top nine active compounds with more corresponding target numbers were quercetin, xanthine, lysine, tyrosine, luteolin, valine, platelet-activating factor, kaempferol, and *ß*-sitosterol, which may be the main compounds of Danlong Dingchuan Decoction in treating asthma, as shown in Table 2.

#### 3.1.4. PPI Network Analysis

**Figure 3:**
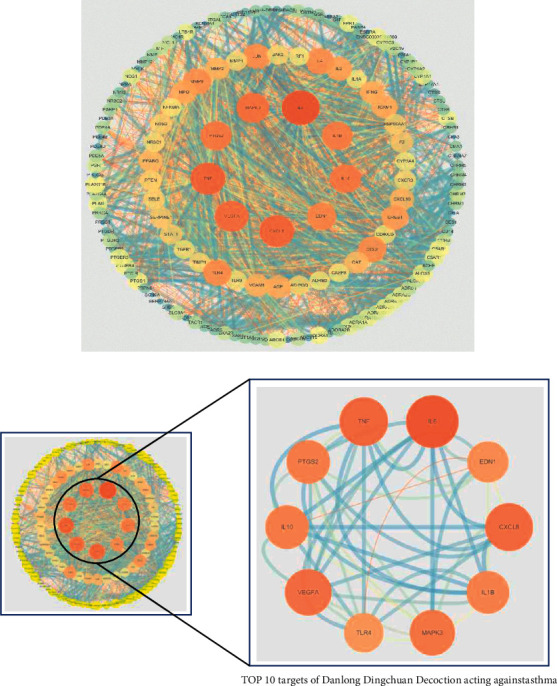
Protein-protein interaction network (PPI network). Circles indicate proteins; the larger the degree value, the larger the circle and the darker the color. The lines represent the nodes associated through PPI; the thicker the line, the closer the relationship between targets.

To show the mechanism by which Danlong Dingchuan Decoction acts on asthma, 155 overlapping targets were imported into the String11.0 platform to obtain the interaction diagram between the targets. The network contained a total of 821 nodes. The results were imported into Cytoscape 3.7.1 software to obtain the PPI network (Figure 3). In the network constructed, the nodes represent targets, the edges represent the interaction relationship between the targets, and the node size and color shade reflect the degree value. As shown in Figure 3, the degrees of targets, such as IL-6, tumor necrosis factor (TNF), IL-8 (CXCL8), vascular endothelial growth factor-A (VEGFA), mitogen-activated protein kinase 3 (MAPK3), IL-10, prostaglandin G/H synthase 2 (PTGS2), IL-1*β*, IL-4, and toll-like receptor 4 (TLR4), were higher. Those targets were related to cell growth, apoptosis, and inflammatory response. Therefore, it was speculated that these targets might be the key targets of Danlong Dingchuan Decoction in treating asthma.

#### 3.1.5. Results of Enrichment Analysis

**Figure 4:**
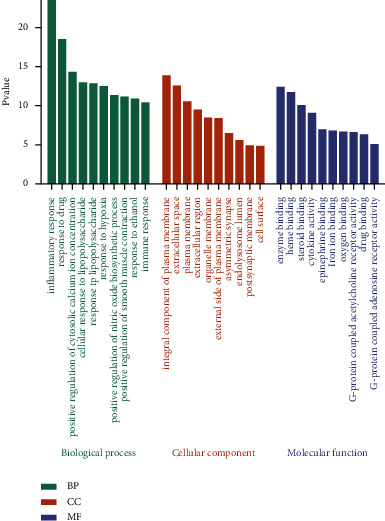
Gene Ontology analysis of the top 10 enrichment results. Green bars represent biological process (BP) analysis; orange bars represent cell component (CC) analysis; and purple bars represent molecular function (MF) analysis.

To further clarify the mechanism by which Danlong Dingchuan Decoction treats asthma, DAVID was used for GO enrichment analysis. A total of 481 items were enriched in the biological process (BP), a total of 39 items were enriched in cell component (CC), and 84 items were enriched in molecular function (MF). With *P* < 0.05 as the screening standard, the top 10 enrichment results were selected for analysis, and each item was presented using a bar chart (Figure 4). The BP was mainly related to the positive regulation of RNA polymerase II promoter transcription, and the MF was closely related to protein binding.

**Figure 5:**
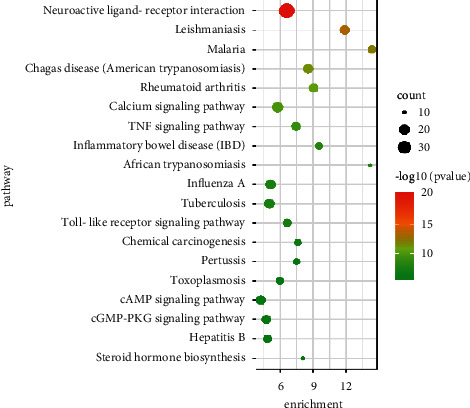
Kyoto Encyclopedia of Genes and Genomes (KEGG) pathway enrichment. The *x*-axis represents the multiple of enrichment; the *y*-axis represents the name of the pathway; the color of the circles in the figure from green to red indicates that the *P* value increases from small to large; and circle sizes from small to large represent the number of genes from fewer to more. The circle size represents the count.

A total of 99 signaling pathways were obtained by KEGG pathway enrichment analysis. The main pathways of Danlong Dingchuan Decoction effects on asthma were as follows: cAMP signaling pathway (fold enrichment = 4.25), cGMP-PKG signaling pathway (fold enrichment = 4.74), NF-*κ*B signaling pathway (fold enrichment = 5.92), and PI3K-Akt signaling pathway (fold enrichment = 2.17), which were associated with MAPK3, TLR4, TNF, and other similar proteins (Figure 5). The top 20 pathways were selected by sorting the *P*-value in ascending order, and the enrichment pathway was visually displayed by the enrichment bubble graph.

#### 3.1.6. Molecular Docking of the Potential Targets with the Compound Components

**Figure 6:**
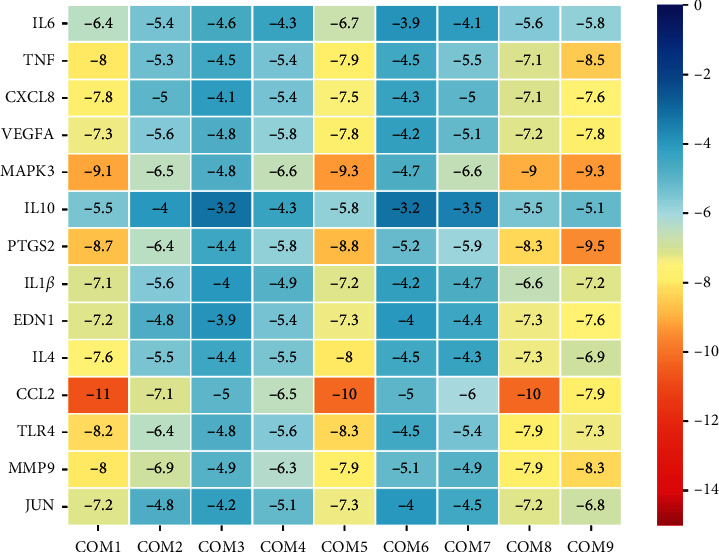
Heatmap of molecular docking results. The *x*-axis represents the bioactive compounds; *y*-axis represents the key targets. COM1 = quercetin; COM2 = xanthine; COM3 = lysine; COM4 = tyrosine; COM5 = luteolin; COM6 = valine; COM7 = platelet-activating factor; COM8 = kaempferol; COM9 = *ß*-sitosterol.

**Figure 7:**
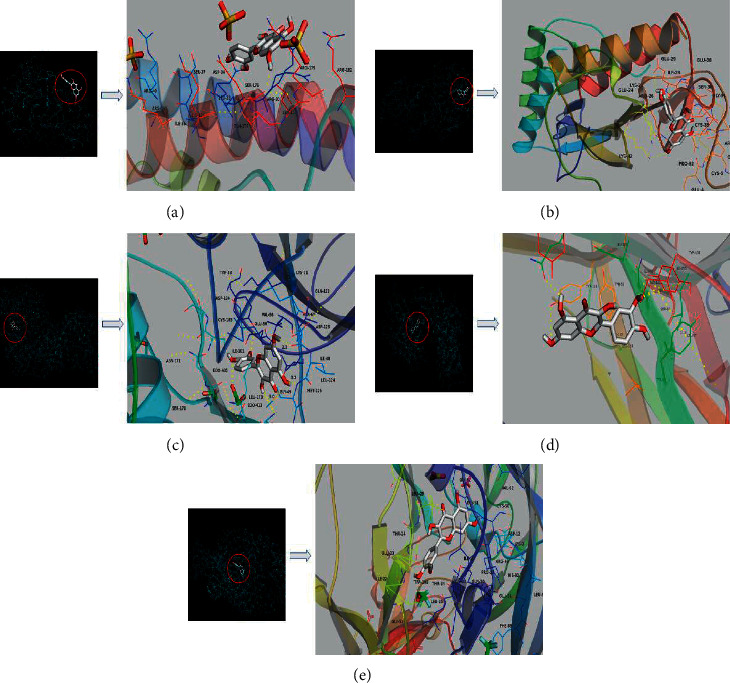
The protein-ligand of the docking simulation: (a) quercetin and IL-6; (b) quercetin and CXCL8; (c) quercetin and MAPK3; (d) quercetin and TNF; (e) quercetin and VEGFA.

AutoDockTools 1.5.6 was used to conduct molecular docking between the potential targets of Danlong Dingchuan Decoction acting on asthma and the main compounds (quercetin, xanthine, lysine, tyrosine, luteolin, valine, platelet-activating factor, kaempferol, and *ß*-sitosterol) determined by topology analysis. The higher absolute value of a docking result indicates a stronger binding force between the active site of the protein receptor and the compound. The more stable the conformation of the ligand binding to the receptor is, the more likely it is to act. Docking scores are shown in Figure 6. Quercetin, which may be the main bioactive compound of Danlong Dingchuan Decoction, had the largest number of target points, and its docking effects with the key targets are shown in Figure 7.

### 3.2. Experimental Results

#### 3.2.1. Danlong Dingchuan Decoction Reduces Tracheal Inflammatory Cell Infiltration in the Lung Tissue of Asthmatic Mice

**Figure 8:**
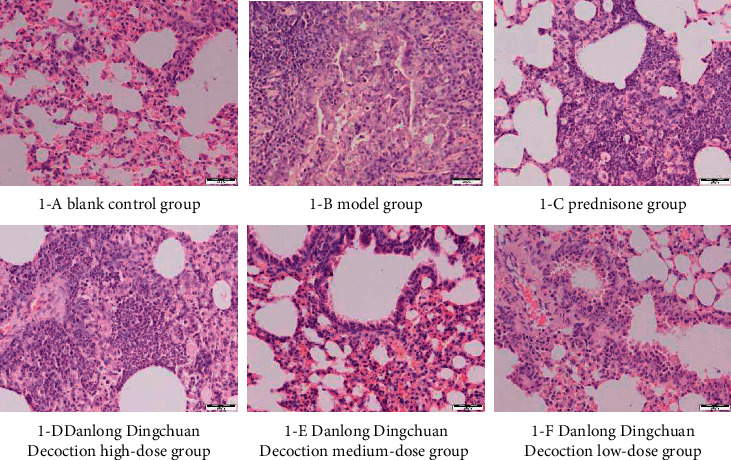
Histopathological appearance of the lung tissue in each group after 42 days of decoction intervention (H&E, 200×).

H&E staining results showed that the normal group mice had normal bronchial structure, intact airway epithelium, and no obvious inflammatory cell infiltration, whereas the other groups had different degrees of pathological changes in the bronchial structure. Mice in the model group had thickened airway wall, narrowed lumen, and increased airway mucosal folds, with exfoliation and necrotic epithelial cells, smooth muscle spasm, thickened basement membrane of airway smooth muscle, and a large number of neutrophils and lymphocytes that had infiltrated the submucosa and tube wall. The pathological changes in the prednisone and Danlong Dingchuan Decoction groups were lighter than those in the model group (Figure 8).

#### 3.2.2. Danlong Dingchuan Decoction Reduces TNF-*α*, IL-4, TGF-*β*1, IL-6, IL-8, and IL-1*β* Expression Levels in the Lung Tissue of Asthmatic Mice

According to the network pharmacology prediction results, Danlong Dingchuan Decoction was closely related to inflammation in treating asthma. Therefore, the expression levels of TNF-*α,* IL-4, TGF-*β*1, IL-6, IL-8, and IL-1*β* in the lung tissue were detected in OVA-induced asthmatic mice models. Compared with the blank group, the expression levels of TNF-*α*, IL-4, TGF-*β*1, IL-6, IL-8, and IL-1*β* in the lung tissue of asthmatic mice were significantly increased, *∗p* < 0.05. Compared with the model group, Danlong Dingchuan Decoction effectively reduced the expression levels of TNF-*α*, IL-4, TGF-*β*1, IL-6, IL-8, and IL-1*β* in the lung tissue, reduced the release of inflammatory factors, alleviated airway inflammatory response, and relieved asthma symptoms (Table 3).

#### 3.2.3. Effects of Danlong Dingchuan Decoction on TLR4 mRNA Expression in the Lung Tissue of Asthmatic Mice

The PPI and KEGG analyses indicated that TLR4 might be one of the targets of Danlong Dingchuan Decoction in treating asthma, as further confirmed by the molecular docking results. Therefore, TLR4 mRNA expression was detected in the lung tissue of asthmatic mice. Compared with the blank control group, TLR4 mRNA was increased in the model group, but the difference was not statistically significant *p* > 0.05. TLR4 mRNA was reduced in the high-dose and low-dose Danlong Dingchuan Decoction groups, but the differences were not statistically significant, *p* > 0.05. TLR4 mRNA in the medium-dose Danlong Dingchuan Decoction group showed no significant change.

## 4. Discussion

TCM theory believes that the etiology of asthma disease is first responsible for the deficiency of the lungs, spleen, and kidneys. Abnormal water metabolism leads to phlegm and then forms stubborn sputum, which lurks in the lungs. Moreover, the lung's qi (or Zong qi) is insufficient, whereas blood flow is not smooth; as a result, phlegm and blood stasis become cemented. When this phenomenon lasts for a long time, it gradually leads to the lack of nourishment of the airways, eventually damaging the function of the lungs, which is known as the deficiency of “lung's yang.” Therefore, “deficiency,” “phlegm,” and “blood stasis” are the main pathogenetic factors of asthma, and throughout the whole pathogenesis process, “tonifying deficiency, eliminating phlegm, and removing blood stasis” are the basic strategies of TCM against asthma [12, 13].

Danlong Dingchuan Decoction is a commonly used clinical decoction for treating asthma based on Jinshui Liujun Decoction in “Jingyue Quanshu · Xinfang Bazhen.” The formula contains Erchen Decoction, Faxia, Chenpi, Fuling, and Gancao to strengthen the spleen and reduce phlegm; it also contains Danshen and Dilong to promote blood circulation and remove stasis while ventilating collaterals and relieving asthma, aiming at the pathological characteristics of “phlegm and stasis interlaced” in asthma. Besides, Danggui, Shudihuang, and Baishao are added for nourishing yin and blood, tonifying deficiency of yin and blood of the lungs and kidneys, nourishing kidney water to protect lung gold, playing the effects of tonifying qi and strengthening body resistance, promoting blood circulation and dredging collaterals, and resolving phlegm and relieving asthma while considering the three aspects of tonifying deficiency, eliminating phlegm, and removing blood stasis. To explore the potential mechanism by which Danlong Dingchuan Decoction treats asthma, a “herb-compound-target-disease” network was constructed by the network pharmacology method. This study indicated that Danlong Dingchuan Decoction contains a variety of bioactive compounds that may protect against asthma through a variety of pathways and targets.

**Figure 9:**
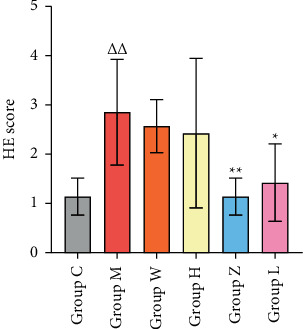
H&E staining scores for inflammation degree. *Note.* Group C: blank control group; Group M: model group; Group W: prednisone group; Group H: Danlong Dingchuan Decoction high-dose group; Group Z: Danlong Dingchuan Decoction medium-dose group; Group L: Danlong Dingchuan Decoction low-dose group. Compared with the blank control group, ΔΔP < 0.01 and ΔP < 0.05; compared with the model group, ^*∗∗*^*p* < 0.01and ^*∗*^*p* < 0.05.

Asthma is a chronic inflammatory disease of the airways that involves multiple cells and cellular components. The chronic inflammation leads to airway hyperresponsiveness, besides irreversible airway stenosis and remodeling with the prolongation of the disease course. Therefore, chronic airway inflammation and airway remodeling are the two main pathological features of asthma. Eliminating airway inflammation and preventing airway remodeling are key approaches in treating asthma. As shown in the H&E staining, airway inflammatory cell infiltration was obvious in the pathological sections of the lung tissue in the asthmatic mice model, and inflammatory cell infiltration was decreased in all doses of Danlong Dingchuan Decoction groups compared with the model group, indicating that Danlong Dingchuan Decoction had a positive effect on alleviating the inflammatory state of asthma. However, the alleviation of airway inflammation was more obvious in the medium- and low-dose Danlong Dingchuan Decoction groups, which may be related to the complex metabolic dynamics of the TCM compound (Figure 9).

Th1 and Th2 cells are two different subgroups of CD4+ Th cells, and CD4+ Th cells are important immune cells in the human body that participate in the immune response and play an important role in the pathogenesis of asthma [14]. Among them, Th2 cells are crucial for lung mucosal immunity. Therefore, according to classical immune theories, the imbalance in both the number and the function of Th1 and Th2 cell subgroups, especially the overactivity of Th2 cytokines, often leads to a chronic inflammatory airway disease, asthma [15]. In other words, the complex inflammatory process that is formed by cytokines, other inflammatory mediators, and inflammatory cells, maintaining the airway in an inflammatory response state dominated by Th2 cytokines, is the pathological basis of asthma. Based on the abovementioned theory, it was hypothesized that Danlong Dingchuan Decoction could inhibit Th2-mediated asthma by affecting Th1/Th2 cell balance and decreasing Th2 cytokine expression.

This study suggested that quercetin, xanthine, lysine, tyrosine, luteolin, valine, platelet-activating factor, kaempferol, and *ß*-sitosterol are the predicted main active compounds, and they may be the main compounds of Danlong Dingchuan Decoction in the treatment of asthma. All the predicted compounds mentioned above can be found in 11 herbs contained in Danlong Dingchuan Decoction, which is consistent with the literature reports [16–39] (Table 4). However, how the predicted compounds work still needs further study, which is one of the directions of our future research.

According to the prediction results of network pharmacology, IL-6, TNF, CXCL8, IL-10, IL-1*β*, and IL-4 may be the key targets of Danlong Dingchuan Decoction against asthma. Among them, IL-4, IL-6, and IL-8 are Th2 cytokines [40]. When Th2 cytokine secretion increases, it promotes IgE production, stimulating the proliferation and activation of eosinophils and secreting various inflammatory mediators, thereby causing chronic airway inflammation [41–43]. IL-1*β*, the main secretory isomer of IL-1, mediates the development of asthma by differentiating and activating Th2 and Th17 cells [44]. Our experimental results showed that Danlong Dingchuan Decoction could effectively reduce IL-4, IL-6, IL-8, and IL-1*β* expression in the lung tissue of asthmatic mice and inhibit the inflammatory response state mediated by Th2 cytokines. TGF-*β*1 is an isomer of TGF-*β* that can combine with IL-6 to mediate the secretion of IL-17 by Th17 under IL-23 stimulation and induce airway inflammation [45]. Danlong Dingchuan Decoction can also decrease TGF-*β*1 expression, decrease IL-17 secretion, and reduce airway inflammation, which is consistent with the expected hypothesis that the mechanism of Danlong Dingchuan Decoction in treating asthma includes alleviating airway inflammation, reducing the expression of Th2 cytokines, affecting the balance of Th1/Th2 cells, and thus inhibiting Th2-mediated asthma.

TNF-*α* is the main cell surface molecule necessary for dendritic cells to produce IL-12, the polarization cytokine of Th1 [1]. However, Danlong Dingchuan Decoction also reduced TNF-*α* expression in the lung tissue of asthmatic mice, which is inconsistent with the hypothesis. The inhibition of TNF-*α* expression in the lung tissue of mice by Danlong Dingchuan Decoction may be related to the complexity of Chinese herbal medicine and formulas, but its specific mechanism remains to be further studied.

TLR4 expression by lung cells (probably lung epithelial cells) is necessary and sufficient for Th2 cell differentiation. After activation through TLR4, lung epithelial cells produce TSLP, IL-25, and IL-33, which are important cytokines for Th2 cells to function [46]. Therefore, it was hypothesized that decreased TLR4 expression might affect Th2 cell differentiation and decrease Th2 cytokine level, which may affect Th1/Th2 cytokine balance. Unfortunately, although the changes in TLR4 mRNA levels in each group were measured in this experiment, the increase in TLR4 mRNA in the model group was not statistically significant compared with the blank control group. Compared with the model group, there was no statistical significance in the decrease of TLR4 mRNA in the low- and high-dose Danlong Dingchuan Decoction groups. TLR4 mRNA in the medium-dose Danlong Dingchuan Decoction group had no significant change. The reasons may be related to small sample capacity, selection of experimental mice, selection of experimental drug's type and dosage forms, or individual differences in the response of the mice to experimental drugs. The specific mechanism still needs to be further studied in larger samples.

In addition, according to the network pharmacology analysis, quercetin and *ß*-sitosterol had a strong binding ability to TNF, so they may be potential bioactive compounds of Danlong Dingchuan Decoction in treating asthma. It has been confirmed that quercetin can reduce airway inflammation by downregulating IL-4, IL-5, and miR-155 levels [47]. In contrast, *ß*-sitosterol can inhibit the expression of inflammatory cytokine TNF-*α* and play an antiasthmatic role by inhibiting cellular response and Th2 cytokine release [14].

Our KEGG analysis showed that the cAMP and NF-*κ*B signaling pathways were related to Danlong Dingchuan Decoction in treating asthma. cAMP-related signaling pathways are involved in the inflammatory response, exudation, and fibrosis and are common signaling pathways for TCM to exert anti-inflammatory effects [15]. The NF-*κ*B signaling pathway, the most important downstream inflammatory signaling pathway of cAMP, could further inhibit the release of proinflammatory factors by decreasing the activity of TLR4 and reducing the activity of NF-*κ*B and Th2 factors, such as TNF-*α*, IL-6, IL-4, and IL-5 in asthmatic mice [48], thus alleviating asthma. The experimental results confirmed that Danlong Dingchuan Decoction could reduce TLR4 mRNA expression in asthmatic mice. Therefore, TLR4 may be one of the targets of Danlong Dingchuan Decoction in treating asthma. As mentioned above, it can be speculated that Danlong Dingchuan Decoction could reduce asthmatic inflammation by reducing TLR4 mRNA levels and regulating NF-*κ*B signaling pathway expression.

**Figure 10:**
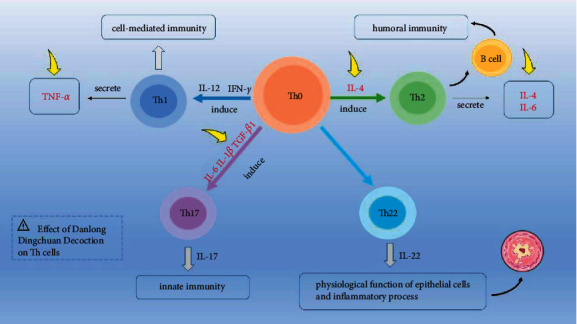
Effect of Danlong Dingchuan Decoction on Th cells. Yellow arrows point to the targets of Danlong Dingchuan Decoction acting on Th cytokines.

In conclusion, Danlong Dingchuan Decoction may act on IL-4, IL-6, IL-8, and other Th2 cytokines and reduce the expression of Th2 cytokines with a variety of active compounds in treating asthma (Figure 10). This study tried to uncover the pharmacology of Danlong Dingchuan Decoction in treating asthma based on the network pharmacology approach. This was in line with the relevant content of Network Pharmacology Evaluation Method Guidance [49] and provided a reference for further studies. However, the composition of TCM prescription is strictly regulated, and the mechanism by which it affects disease is quite complex. TCM action is not only based on simply adding herbs together or adding the active compounds contained in the herbs together. Every single herb contains varieties of active compounds. Although the same herb is used in different decoctions, it still has different active compounds working to treat different diseases. In addition, network pharmacology largely relies on network databases to obtain original data, so there are situations wherein the results obtained by network pharmacology are inconsistent with expectations or cannot be verified by experiments. Therefore, in the future study, we plan to conduct a further study on the main compounds or the related signaling pathways of Danlong Dingchuan Decoction to compensate for the limitations of this experiment and to provide new ideas for better revealing the mechanisms by which TCM acts with multiple compounds, targets, and pathways.

## 5. Conclusion

According to the network pharmacology results, quercetin, xanthine, lysine, tyrosine, luteolin, valine, platelet-activating factor, kaempferol, and *ß*-sitosterol are the main active components, and IL-6, TNF, CXCL8, VEGFA, MAPK3, IL-10, PTGS2, IL-1*β*, IL-4, and TLR4 are the potential targets of Danlong Dingchuan Decoction in treating asthma. cAMP and NF-*κ*B signaling pathways may play an important role in treating asthma. The animal experiments verified that Danlong Dingchuan Decoction could effectively reduce IL-4, IL-6, IL-8, and IL-1*β* expression in the lung tissue of asthmatic mice, decrease TLR4 mRNA expression, and inhibit Th2 cytokine-mediated inflammatory response to treat asthma.

## Figures and Tables

**Table 1:** Primer sequences of TLR4.

Name of the primer	The sequence of the primer (5′ to 3′)
TLR4-F	CACTGCATGTGACTTTCCTTGATT
TLR4-R	CACTCAGACTCGGCACTTAGCA

**Table 2:** Basic information of main compounds of Danlong Dingchuan Decoction.

Compound	Chemical component	Number of targets	Herbs containing the compound
1	Quercetin	144	Kuandonghua; Gancao
2	Xanthine	92	Dilong
3	Lysine	68	Dilong
4	Tyrosine	63	Dilong
5	Luteolin	55	Danshen; Shegan
6	Valine	48	Dilong
7	Platelet-activating factor	44	Dilong
8	Kaempferol	36	Baishao; Kuandonghua; Gancao
9	*β*-Sitosterol	35	Danggui; Fabanxia; Baishao; Kuandonghua

**Table 3:** Effect of Danlong Dingchuan Decoction on the concentrations of TNF-*α*, IL-4, TGF-*β*1, IL-6, IL-8, and IL-1*β* in the lung tissue of asthmatic mice ($\bar{x}$± *s*, ng • mL^−1^, *n* = 10).

Group	TNF-*α* (ng • mL^−1^)	IL-4 (ng • mL^−1^)	TGF-*β*1 (ng • mL^−1^)	IL-6 (ng • mL^−1^)	IL-8 (ng • mL^−1^)	IL-1*β* (ng • mL^−1^)
C	32.08 ± 6.36	36.05 ± 7.67	6077.89 ± 2287.15	44.97 ± 12.17	198.57 ± 46.36	50.31 ± 5.27
M	40.00 ± 4.05^∆∆^	63.26 ± 14.99^∆∆^	11397.99 ± 1785.00^∆∆^	81.13 ± 18.99^△^	438.25 ± 164.31^∆^	136.06 ± 43.97^∆^
W	32.74 ± 5.55^*∗∗*^	37.88 ± 10.89^*∗∗*^	7109.92 ± 1398.58^*∗∗*^	63.56 ± 18.37^∗^	241.52 ± 41.25^*∗*^	56.12 ± 12.16^*∗*^
H	33.00 ± 3.23^*∗∗*^	60.37 ± 14.05	1700.25 ± 1569.72	47.69 ± 9.85^*∗*^	471.01 ± 163.81	48.01 ± 10.24^*∗*^
Z	32.52 ± 6.23^*∗∗*^	55.63 ± 9.11	11006.92 ± 1569.29	44.12 ± 9.00^*∗*^	253.13 ± 98.56	85.82 ± 54.60
L	31.96 ± 3.11^*∗∗*^	42.44 ± 12.847^*∗∗*^	7536.31 ± 1551.97^*∗∗*^	66.30 ± 17.15^*∗*^	319.23 ± 71.05^*∗*^	543.48 ± 193.11^*∗*^

Group C: blank control group; Group M: model group; Group W: prednisone group; Group H: Danlong Dingchuan Decoction high-dose group; Group Z: Danlong Dingchuan Decoction medium-dose group; Group L: Danlong Dingchuan Decoction low-dose group. Compared with the blank control group, ^ΔΔ^*P* < 0.01 and ^Δ^*p* < 0.05; compared with the model group, ^*∗∗*^*p* < 0.01 and ^*∗*^*p* < 0.05.

**Table 4:** Main active compounds contained in 11 herbs in Danlong Dingchuan Decoction.

Herb	Containing compounds
Danggui	Beta-sitosterol [16]
Danshen	Beta-sitosterol [17]
Danshen	Quercetin [18]
Danshen	Kaempferol [18]
Shudihuang	Tyrosine [19, 20]
Shudihuang	Valine [19, 20]
Dilong	Xanthine [21–23]
Dilong	Lysine [21–23]
Dilong	Tyrosine [21–23]
Dilong	Valine [21–23]
Dilong	Platelet-activating factor [22]
Fabanxia	Xanthine [24]
Fabanxia	Lysine [25–27]
Fabanxia	Tyrosine [25–27]
Fabanxia	Valine [25–27]
Fabanxia	Beta-sitosterol [25, 27]
Chenpi	Beta-sitosterol [28, 29]
Gancao	Quercetin [30]
Gancao	Kaempferol [30]
Gancao	Beta-sitosterol [30]
Fuling	Lysine [31]
Fuling	Tyrosine [31]
Fuling	Beta-sitosterol [31]
Shegan	Quercetin [32]
Shegan	Luteolin [32, 33]
Shegan	Kaempferol [32, 34]
Shegan	Beta-sitosterol [32, 33]
Kuandonghua	Quercetin [35]
Kuandonghua	Luteolin [36]
Kuandonghua	Kaempferol [36]
Kuandonghua	Beta-sitosterol [36]
Baishao	Kaempferol [37–39]

## Data Availability

All the data can be obtained from the open-source platform provided in the paper, and conclusions can be drawn through analyses in the relevant software programs.

## Supplementary Materials

Supplementary Materials Table S1: basic information on active compounds in Danlong Dingchuan Decoction. Table S2: gene targets of Danlong Dingchuan Decoction and asthma. Figure S1: graphical abstract of the paper.
