# Supplementary material for: Network Pharmacology Combined with Molecular Docking and Experimental Verification Reveals the Bioactive Components and Potential Targets of Danlong Dingchuan Decoction against Asthma
Source: Evid Based Complement Alternat Med. 2022 Feb 10;2022:7895271. doi: 10.1155/2022/7895271 (PMC8853800; doi:10.1155/2022/7895271)
Supplement: Supplementary Materials — Table S1: basic information on active compounds in Danlong Dingchuan Decoction. Table S2: gene targets of Danlong Dingchuan Decoction and asthma. Figure S1: graphical abstract of the paper. [file 7895271.f1.zip › 7895271.f1/Supplementary Materials-Table S1.pdf]

Herb	MOLID	Active Compounds
herb1	MOL000006	luteolin
herb1	MOL000569	digallate
herb1	MOL001601	1,2,5,6-tetrahydrotanshinone
herb1	MOL001659	Poriferasterol
herb1	MOL001771	poriferast-5-en-3beta-ol
herb1	MOL001942	isoimperatorin
herb1	MOL002222	sugiol
herb1	MOL002651	Dehydrotanshinone II A
herb1	MOL002776	Baicalin
herb1	MOL007036	5,6-dihydroxy-7-isopropyl-1,1-dimethyl-2,3-dihydroph[illegible]
herb1	MOL007041	2-isopropyl-8-methylphenanthrene-3,4-dione
herb1	MOL007045	3α-hydroxytanshinone Ⅱ a
herb1	MOL007048	(E)-3-[2-(3,4-dihydroxyphenyl)-7-hydroxy-benzofuran-[illegible]
herb1	MOL007049	4-methylenemiltirone
herb1	MOL007050	2-(4-hydroxy-3-methoxyphenyl)-5-(3-hydroxypropyl)-[illegible]
herb1	MOL007058	formyltanshinone
herb1	MOL007059	3-beta-Hydroxymethyllenetanshiquinone
herb1	MOL007061	Methylenetanshinquinone
herb1	MOL007063	przewalskin a
herb1	MOL007064	przewalskin b
herb1	MOL007068	Przewaquinone B
herb1	MOL007069	przewaquinone c
herb1	MOL007070	(6S,7R)-6,7-dihydroxy-1,6-dimethyl-8,9-dihydro-7H-na[illegible]
herb1	MOL007071	przewaquinone f
herb1	MOL007077	sclareol
herb1	MOL007079	tanshinaldehyde
herb1	MOL007081	Danshenol B
herb1	MOL007082	Danshenol A
herb1	MOL007085	Salvilenone
herb1	MOL007088	cryptotanshinone
herb1	MOL007093	dan-shexinkum d
herb1	MOL007094	danshenspiroketallactone
herb1	MOL007098	deoxyneocryptotanshinone
herb1	MOL007100	dihydrotanshinlactone
herb1	MOL007101	dihydrotanshinone Ⅰ
herb1	MOL007105	epidanshenspiroketallactone
herb1	MOL007107	C09092
herb1	MOL007108	isocryptotanshi-none
herb1	MOL007111	Isotanshinone II
herb1	MOL007115	manool
herb1	MOL007119	miltionone Ⅰ
herb1	MOL007120	miltionone Ⅱ
herb1	MOL007121	miltipolone
herb1	MOL007122	Miltirone
herb1	MOL007124	neocryptotanshinone ii
herb1	MOL007125	neocryptotanshinone
herb1	MOL007127	1-methyl-8,9-dihydro-7H-naphtho[5,6-g]benzofuran-6,[illegible]
herb1	MOL007130	prolithospermic acid
herb1	MOL007132	(2R)-3-(3,4-dihydroxyphenyl)-2-[(Z)-3-(3,4-dihydroxy[illegible]
herb1	MOL007141	salvianolic acid g
herb1	MOL007142	salvianolic acid j
herb1	MOL007143	salvilenone Ⅰ
herb1	MOL007145	salviolone
herb1	MOL007150	(6S)-6-hydroxy-1-methyl-6-methylol-8,9-dihydro-7H-n[illegible]
herb1	MOL007151	Tanshindiol B
herb1	MOL007152	Przewaquinone E
herb1	MOL007154	tanshinone iia

herb1	MOL007155	(6S)-6-(hydroxymethyl)-1,6-dimethyl-8,9-dihydro-7H-n
herb1	MOL007156	tanshinone Ⅵ
herb2	MOL000358	beta-sitosterol
herb2	MOL000449	stigmasterol
herb3	MOL000359	sitosterol
herb3	MOL000449	stigmasterol
herb4	MOL000358	beta-sitosterol
herb4	MOL000449	Stigmasterol
herb4	MOL000519	coniferin
herb4	MOL001755	24-Ethylcholest-4-en-3-one
herb4	MOL002670	Cavidine
herb4	MOL002714	baicalein
herb4	MOL002776	Baicalin
herb4	MOL003578	Cycloartenol
herb4	MOL005030	gondoic acid
herb4	MOL006936	10,13-eicosadienoic
herb4	MOL006957	(3S,6S)-3-(benzyl)-6-(4-hydroxybenzyl)piperazine-2,5-
herb4	MOL006967	beta-D-Ribofuranoside, xanthine-9
herb5	MOL000359	sitosterol
herb5	MOL004328	naringenin
herb5	MOL005100	5,7-dihydroxy-2-(3-hydroxy-4-methoxyphenyl)chromar
herb5	MOL005815	Citromitin
herb5	MOL005828	nobiletin
herb6	MOL000273	(2R)-2-[(3S,5R,10S,13R,14R,16R,17R)-3,16-dihydroxy-4,4,10,13,14-pentamethyl-2,3,5,6,12,15,16,17-octahydro-1H-cyclopenta[a]phenanthren-17-yl]-6-methylhept-5-enoic acid
herb6	MOL000275	trametenolic acid
herb6	MOL000279	Cerevisterol
herb6	MOL000282	ergosta-7,22E-dien-3beta-ol
herb6	MOL000283	Ergosterol peroxide
herb6	MOL000296	hederagenin
herb7	MOL000006	luteolin
herb7	MOL000351	Rhamnazin
herb7	MOL000354	isorhamnetin
herb7	MOL000449	Stigmasterol
herb7	MOL001735	Dinatin
herb7	MOL002322	isovitexin
herb7	MOL003742	ardisianone A
herb7	MOL003753	dihydrokaempferide
herb7	MOL003758	Iristectorigenin (9CI)
herb7	MOL003759	Iristectorigenin A
herb7	MOL003769	Irolone
herb8	MOL000098	quercetin
herb8	MOL000358	beta-sitosterol
herb8	MOL000422	kaempferol
herb8	MOL010003	7beta-angeloyloxyoplopa-3(14)Z,8(10)-dien-2-one
herb8	MOL010004	7beta-(4-methylsenecioyloxy)oplopa-3(14)E,8(10)-dien
herb8	MOL010006	7beta-senecioyloxyoplopa-3(14)Z,8(10)-dien-2-one
herb8	MOL010007	la-7beta-di(4-methulsenecioyloxy) oplopa-3(14)Z,8(10)
herb8	MOL010013	7beta-(3-ethyl-cis-crotonoyloxy)-14-hydroxy-notonipet
herb8	MOL010014	14-acetoxy-7beta-angeloyloxy-notonipetranone
herb8	MOL010015	14-acetoxy-7beta-senecioyloxy-notonipetranone
herb8	MOL010017	7beta-(3-ethyl-cis-crotonoyloxy)-la-(2-methyl butyrylo:
herb8	MOL010023	senkirkine
herb8	MOL010028	tussilagin
herb8	MOL010055	Femara

herb8	MOL010058	methyl 3-o-caffeoylquinate
herb8	MOL010060	Methyl butyric acid tussilagin ester
herb9	MOL000211	Mairin
herb9	MOL000358	beta-sitosterol
herb9	MOL000359	sitosterol
herb9	MOL000422	kaempferol
herb9	MOL000492	(+)-catechin
herb9	MOL001918	paeoniflorgenone
herb9	MOL001919	(3S,5R,8R,9R,10S,14S)-3,17-dihydroxy-4,4,8,10,14-pe
herb9	MOL001924	paeoniflorin
herb10	MOL001484	Inermine
herb10	MOL001792	DFV
herb10	MOL000211	Mairin
herb10	MOL002311	Glycyrol
herb10	MOL000239	Jaranol
herb10	MOL002565	Medicarpin
herb10	MOL000354	isorhamnetin
herb10	MOL000359	sitosterol
herb10	MOL003656	Lupiwighteone
herb10	MOL003896	7-Methoxy-2-methyl isoflavone
herb10	MOL000392	formononetin
herb10	MOL000417	Calycosin
herb10	MOL000422	kaempferol
herb10	MOL004328	naringenin
herb10	MOL004805	(2S)-2-[4-hydroxy-3-(3-methylbut-2-enyl)phenyl]-8,8-d
herb10	MOL004806	euchrenone
herb10	MOL004808	glyasperin B
herb10	MOL004810	glyasperin F
herb10	MOL004811	Glyasperin C
herb10	MOL004814	Isotrifoliol
herb10	MOL004815	(E)-1-(2,4-dihydroxyphenyl)-3-(2,2-dimethylchromen-6
herb10	MOL004820	kanzonols W
herb10	MOL004824	(2S)-6-(2,4-dihydroxyphenyl)-2-(2-hydroxypropan-2-yl
herb10	MOL004827	Semilicoisoflavone B
herb10	MOL004828	Glepidotin A
herb10	MOL004829	Glepidotin B
herb10	MOL004833	Phaseolinisoflavan
herb10	MOL004835	Glypallichalcone
herb10	MOL004838	8-(6-hydroxy-2-benzofuranyl)-2,2-dimethyl-5-chromen
herb10	MOL004841	Licochalcone B
herb10	MOL004848	licochalcone G
herb10	MOL004849	3-(2,4-dihydroxyphenyl)-8-(1,1-dimethylprop-2-enyl)-7
herb10	MOL004855	Licoricone
herb10	MOL004856	Gancaonin A
herb10	MOL004857	Gancaonin B
herb10	MOL004863	3-(3,4-dihydroxyphenyl)-5,7-dihydroxy-8-(3-methylbut
herb10	MOL004864	5,7-dihydroxy-3-(4-methoxyphenyl)-8-(3-methylbut-2-e
herb10	MOL004866	2-(3,4-dihydroxyphenyl)-5,7-dihydroxy-6-(3-methylbut
herb10	MOL004879	Glycyrin
herb10	MOL004882	Licocoumarone
herb10	MOL004883	Licoisoflavone
herb10	MOL004884	Licoisoflavone B
herb10	MOL004885	licoisoflavanone
herb10	MOL004891	shinpterocarpin
herb10	MOL004898	(E)-3-[3,4-dihydroxy-5-(3-methylbut-2-enyl)phenyl]-1-
herb10	MOL004903	liquiritin
herb10	MOL004904	licopyranocoumarin
herb10	MOL004907	Glyzaglabrin

herb10	MOL004908	Glabridin
herb10	MOL004910	Glabranin
herb10	MOL004911	Glabrene
herb10	MOL004912	Glabrone
herb10	MOL004913	1,3-dihydroxy-9-methoxy-6-benzofurano[3,2-c]chrome
herb10	MOL004914	1,3-dihydroxy-8,9-dimethoxy-6-benzofurano[3,2-c]chro
herb10	MOL004915	Eurycarpin A
herb10	MOL004924	(-)-Medicocarpin
herb10	MOL004935	Sigmoidin-B
herb10	MOL004941	(2R)-7-hydroxy-2-(4-hydroxyphenyl)chroman-4-one
herb10	MOL004945	(2S)-7-hydroxy-2-(4-hydroxyphenyl)-8-(3-methylbut-2-
herb10	MOL004948	Isoglycyrol
herb10	MOL004949	Isolicoflavonol
herb10	MOL004957	HMO
herb10	MOL004959	1-Methoxyphaseollidin
herb10	MOL004961	Quercetin der.
herb10	MOL004966	3'-Hydroxy-4'-O-Methylglabridin
herb10	MOL000497	licochalcone a
herb10	MOL004974	3'-Methoxyglabridin
herb10	MOL004978	2-[(3R)-8,8-dimethyl-3,4-dihydro-2H-pyrano[6,5-f]chro
herb10	MOL004980	Inflacoumarin A
herb10	MOL004985	icos-5-enoic acid
herb10	MOL004988	Kanzonol F
herb10	MOL004989	6-prenylated eriodictyol
herb10	MOL004990	7,2',4'-trihydroxy − 5-methoxy-3 − arylcoumarin
herb10	MOL004991	7-Acetoxy-2-methylisoflavone
herb10	MOL004993	8-prenylated eriodictyol
herb10	MOL004996	gadelaidic acid
herb10	MOL000500	Vestitol
herb10	MOL005000	Gancaonin G
herb10	MOL005001	Gancaonin H
herb10	MOL005003	Licoagrocarpin
herb10	MOL005007	Glyasperins M
herb10	MOL005008	Glycyrrhiza flavonol A
herb10	MOL005012	Licoagroisoflavone
herb10	MOL005016	Odoratin
herb10	MOL005017	Phaseol
herb10	MOL005018	Xambioona
herb10	MOL005020	dehydroglyasperins C
herb10	MOL000098	quercetin

herb11		aspartate
herb11		alanine
herb11		methionine
herb11		guanine
herb11		hypoxanthine
herb11		isoleucine
herb11		threonine
herb11		glutamic acid
herb11		phenylalanine
herb11		histidine
herb11		leucine
herb11		xanthine
herb11		proline
herb11		adenine
herb11		lysine
herb11		tyrosine
herb11		valine

herb11	lecithin
herb11	terrestro-lumbrilysin
herb11	nicotinic acid
herb11	adenosine
herb11	guanosine
herb11	succinic Acid
herb11	9-amino-1,3,9-nonane dicarboxylic acid
herb11	glutarylcarnitine
herb11	platelet activating factor
herb11	(S)-2-{[benzyloxy)carbonyl] amino}-5-ethoxy-5-oxope
herb11	adenylosuccinic acid
herb11	3-adenine-9-yl-2-hydroxypropionic acid
herb11	L-tryptophan
herb11	alanyl phenylalanine
herb11	N-{8-[(3-aminopropyl) aminooctyl}-N-α-propionyl-L-t
herb11	desomedine
herb11	2-[4-(2-ethoxyphenyl)-1-piperazinyl]-N-(2-furyl-methy
herb11	ethyl N-acetylbenzoate
herb11	histaminoyl asparagine
herb11	N-(methoxycarbonyl)-L-phenylalanine
herb11	N-bicyclo[2.2.1]hept-2-yl-2-[4-(2-methoxyphenyl)-1-pi

OB	DL
36.16	0.25
61.85	0.26
38.75	0.36
43.83	0.76
36.91	0.75
45.46	0.23
36.11	0.28
43.76	0.4
40.12	0.75
33.77	0.29
40.86	0.23
44.93	0.44
48.24	0.31
34.35	0.23
62.78	0.4
73.44	0.42
32.16	0.41
37.07	0.36
37.11	0.65
110.32	0.44
62.24	0.41
55.74	0.4
41.31	0.45
40.31	0.46
43.67	0.21
52.47	0.45
57.95	0.56
56.97	0.52
30.38	0.38
52.34	0.4
38.88	0.55
50.43	0.31
49.4	0.29
38.68	0.32
45.04	0.36
68.27	0.31
36.07	0.25
54.98	0.39
49.92	0.4
45.04	0.2
49.68	0.32
71.03	0.44
36.56	0.37
38.76	0.25
39.46	0.23
52.49	0.32
34.72	0.37
64.37	0.31
109.38	0.35
45.56	0.61
43.38	0.72
32.43	0.23
31.72	0.24
75.39	0.46
42.67	0.45
42.85	0.45
49.89	0.4

65.26	0.45
45.64	0.3
36.91	0.75
43.83	0.76
36.91	0.75
43.83	0.76
36.91	0.75
43.83	0.76
31.11	0.32
36.08	0.76
35.64	0.81
33.52	0.21
40.12	0.75
38.69	0.78
30.7	0.2
39.99	0.2
46.89	0.27
44.72	0.21
36.91	0.75
59.29	0.21
47.74	0.27
86.9	0.51
61.67	0.52
30.93	0.81
38.71	0.8
37.96	0.77
43.51	0.72
40.36	0.81
36.91	0.75
36.16	0.25
47.14	0.34
49.6	0.31
43.83	0.76
30.97	0.27
31.29	0.72
44.22	0.25
50.56	0.27
71.55	0.34
63.36	0.34
46.87	0.36
46.43	0.28
36.91	0.75
41.88	0.24
40.35	0.22
69.33	0.22
34.09	0.48
42.32	0.52
75.82	0.33
46.04	0.4
37.7	0.4
44.68	0.46
56.16	0.41
61.12	0.38
66.09	0.2

44.39	0.35
34.28	0.61
55.38	0.78
36.91	0.75
36.91	0.75
41.88	0.24
54.83	0.24
87.59	0.37
43.56	0.53
53.87	0.79
75.18	0.54
32.76	0.18
55.38	0.78
90.78	0.67
50.83	0.29
49.22	0.34
49.6	0.31
36.91	0.75
51.64	0.37
42.56	0.2
69.67	0.21
47.75	0.24
41.88	0.24
59.29	0.21
31.79	0.72
30.29	0.57
65.22	0.44
75.84	0.54
45.56	0.4
31.94	0.42
39.62	0.35
50.48	0.52
60.25	0.63
48.78	0.55
44.72	0.35
64.46	0.34
32.01	0.45
61.6	0.19
58.44	0.38
76.76	0.19
49.25	0.32
59.62	0.43
63.58	0.47
51.08	0.4
48.79	0.45
66.37	0.41
30.49	0.41
44.15	0.41
52.61	0.47
33.21	0.36
41.61	0.42
38.93	0.55
52.47	0.54
80.3	0.73
46.27	0.31
65.69	0.74
80.36	0.65
61.07	0.35

53.25	0.47
52.9	0.31
46.27	0.44
52.51	0.5
48.14	0.43
62.9	0.53
43.28	0.37
40.99	0.95
34.88	0.41
71.12	0.18
36.57	0.32
44.7	0.84
45.17	0.42
38.37	0.21
69.98	0.64
46.45	0.33
43.71	0.57
40.79	0.29
46.16	0.57
36.21	0.52
39.71	0.33
30.7	0.2
32.47	0.89
39.22	0.41
83.71	0.27
38.92	0.26
53.79	0.4
30.7	0.2
74.66	0.21
60.44	0.39
50.1	0.78
58.81	0.58
72.67	0.59
41.28	0.6
57.28	0.49
49.95	0.3
78.77	0.58
54.85	0.87
53.82	0.37
46.43	0.28

Compound CID	Molecular Formula
1825910	C4H7NO4
56-41-7	C3H7NO2
59-51-8	C5H11NO2S
73-40-5	C5H5N5O
68-94-0	C5H4N4O
1864445	C6H13NO2
72-19-5	C4H9NO3
1825973	C5H9NO4
63-91-2	C9H11NO2
71-00-1	C6H9N3O2
61-90-5	C6H13NO2
69-89-6	C5H4N4O2
147-85-3	C5H9NO2
73-24-5	C5H5N5
56-87-1	C6H14N2O2
60-18-4	C9H11NO3
72-18-4	C5H11NO2

8002-43-5	
59-67-6	$C_6H_5NO_2$
5536-17-4	$C_{10}H_{13}N_5O_4$
118-00-3	$C_{10}H_{13}N_5O_5$
110-15-6	$C_4H_6O_4$
	$C_{12}H_{21}NO_6$
	$C_{12}H_{21}NO_6$
	$C_{15}H_{21}NO_7$
ntanoic acid	$C_{15}H_{19}NO_6$
19046-78-7	$C_{14}H_{17}N_5O_8$
	$C_8H_9N_5O_3$
73-22-3	$C_{11}H_{12}N_2O_2$
	$C_{12}H_{16}N_2O_3$
yrosi-namide	$C_{23}H_{40}N_4O_3$
	$C_{20}H_{26}N_4O_2$
l) acetamide	$C_{19}H_{25}N_3O_3$
	$C_{11}H_{13}NO_3$
	$C_{10}H_{13}N_4O_3$
	$C_{12}H_{15}NO_4$
perazinyl]acetamide	

herb1=Danshen(SALVIAE MILTIORRHIZAE RADIX ET RHIZOMA)
herb2=Danggui (ANGELICAE SINENSIS RADIX)
herb3=Shudihuang(REHMANNIAE RADIX PRAEPARATA)
herb4=Fabanxia(PINELLIAE RHIZOMA PRAEPARATUM)
herb5=Chenpi(CITRI RETICULATAE PERICARPIUM)
herb6=Fuling(PORIA)
herb7=Shegan(BELAMCANDAE RHIZOMA)
herb8=Kuandonghua(FARFARAE FLOS)
herb9=Baishao(PAEONIAE RADIX ALBA)
herb10=Gancao(GLYCYRRHIZAE RADIX ET RHIZOMA)
herb11=Dilong(PHERETIMA)
