# Supplementary material for: Network Pharmacology Combined with Molecular Docking and Experimental Verification Reveals the Bioactive Components and Potential Targets of Danlong Dingchuan Decoction against Asthma
Source: Evid Based Complement Alternat Med. 2022 Feb 10;2022:7895271. doi: 10.1155/2022/7895271 (PMC8853800; doi:10.1155/2022/7895271)
Supplement: Supplementary Materials — Table S1: basic information on active compounds in Danlong Dingchuan Decoction. Table S2: gene targets of Danlong Dingchuan Decoction and asthma. Figure S1: graphical abstract of the paper. [file 7895271.f1.zip › 7895271.f1/Supplementary Materials-Table S2.pdf]

herb targets	asthma-related targets	overlapping targets
ADCY2	YKL40	CD40LG
AKT1	VTN	GSTP1
APP	VRR1	ICAM1
AR	VIPR	IFNG
BCL2L1	VIP	IL10
BIRC5	VEGFA	IL2
CASP3	VDR	IL4
CASP7	VCAM1	IL6
CASP9	UTS2R	JUN
CCNB1	USP38	MCL1
CCND1	UGT2B7	MMP1
CD40LG	UGT2B15	MMP2
CDK4	UGT1A9	MMP9
CDKN1A	UGT1A8	NFKBIA
DPP4	UGT1A1	PIK3CG
EGFR	UGRP1	PPARG
ERBB2	TYRO3	PRSS1
GSTP1	TYK2	PTGS1
HMOX1	TTLL3	PTGS2
ICAM1	TSLP	TNF
IFNG	Tryptase	VEGFA
IL10	TRPV1	ADRA1A
IL2	TRBC1	ADRA1B
IL4	TRAF5	ADRA1D
IL6	TPT1	ADRB2
INSR	TPSD1	CHRM1
JUN	TPSB2	CHRM2
MAPK1	TPSAB1	CHRM3
MCL1	TOP2	CHRM4
MDM2	TNIP1	CHRM5
MET	TNFSF14	CHRNA7
MMP1	TNFSF10	PDE3A
MMP2	TNFRSF4	SLC6A4
MMP9	TNFR	NR3C2
NCOA2	TNFA	PGR
NFKBIA	TNF	ADRA2A
NUF2	TNC	ADRA2C
PCNA	TLR9	NOS2
PIK3CG	TLR7	NR3C1
PPARG	TLR6	KCNMA1
PRSS1	TLR4	EDN1
PTGES	TLR3	ADRA2B
PTGS1	TLR2	GABRA2
PTGS2	TLR10	CYP1A1
RB1	TLR1	CYP1A2
RELA	TIMP1	CYP3A4
SLC2A4	TGFB1	NR1I2
TNF	TBXAS1	ADORA1
TOP1	TBXA2R	ADORA2A
TOP2A	TBX21	ADORA2B
TP53	TBET	ADORA3
TYR	TAS2R31	LTA4H
VEGFA	TAS2R14	TACR1
XDH	TAS2R10	CPA3
AKR1B1	TACR3	HRH1
ADRA1A	TACR2	CHIA
ADRA1B	TACR1	ACE

ADRA1D	TAC1	F2
ADRB2	SYK mRNA	SHBG
CA2	SYK	HDAC3
CHRM1	SUV39H1	CTSK
CHRM2	STAT6	PTGDR2
CHRM3	STAT4	ITGAL
CHRM4	STAT1	ALOX5
CHRM5	SRD5A2	FABP4
CHRNA7	SPRR2B	MMP12
DRD1	SPRR2A	TBXAS1
GABRA1	SPRED1	SRD5A2
HTR2A	SPINK5	ADRB1
HTR3A	SOD2	MPO
IGHG1	SOCS3	CTSL
NCOA1	SOCS1	MME
OPRD1	SNCA	MIF
OPRM1	SLCO2B1	FPR1
PDE3A	SLCO1B3	TACR2
RXRA	SLCO1B1	HLA-DRB1
SCN5A	SLCO1A2	NPPA
SLC6A3	SLC6A4	ITGA4
SLC6A4	SLC22A8	MC4R
NR3C2	SLC22A7	CTSB
PGR	SLC22A5	HSD11B1
ACHE	SLC22A4	CTSS
DRD2	SLC22A3	C3AR1
ESR1	SLC22A2	TACR3
PTPN1	SLC22A1	SCN9A
CALM1	SIGLEC5	PDE5A
CCNA2	SHBG	JAK2
CDK2	SFTPD	PDE4D
NOS3	SFTA3	CRHR1
PIM1	SETDB2	NOS1
ADRA2A	SERPINE1	TLR4
ADRA2C	SERPINB4	CYP17A1
ESR2	SERPINB3	TRPV1
GSK3B	SERPINB2	BCHE
MAPK14	SERPINA6	PLAU
NOS2	SERPINA1	CXCR3
NR3C1	SELP	CCR8
KCNH2	SELL	HSP90AA1
KCNMA1	SELE	MYLK
EDN1	SEL	TLR9
STAT3	SCYA11	VDR
CHEK1	SCN9A	PTGER2
CHRNA2	SCN10A	UGT2B7
DRD5	SCGB3A2	SERPINA6
F7	SCGB1A1	G6PD
ADRA2B	SATB1	HSD11B2
GABRA2	SART1	PTGER4
GABRA3	S100P	PTGER3
GABRA5	RUNX3	TBXA2R
GABRA6	ROS	PTGDR
GABRE	ROCK	CMA1
GABRG3	RNASE3	CXCL8
HTR2C	RIC3	GSR
SLC6A2	RASGRP4	HDAC2
HTR1A	RAPGEF3	PTPN6

HTR1B
AHSA1
BCL2
CALCR
CYP1A1
CYP1A2
CYP3A4
ECE1
EDNRA
FASN
FOS
ITGB3
MYC
NPM1
NR1I2
EGLN1
ABAT
CACNA2D1
GABBR2
GABBR1
RNPEP
OAT
TH
KMO
PEPD
KYNU
SLC6A1
FYN
LCK
BHMT2
PNP
GDA
TK1
ADORA1
ADORA2A
ADORA2B
ADORA3
SLC1A1
GRM4
GRM5
GRM8
GRM1
GRM7
GRIK1
LTA4H
GRIK2
GABRR1
GRM3
GRM6
GRM2
SLC6A11
SLC6A13
GRIA1
GRIA4
SLC1A2
PLG
GRIK3
GRIA2
PTPN6
PTK
PTGS2
PTGS1
PTGIR
PTGER4
PTGER3
PTGER2
PTGDR2
PTGDR
PTEN
PTAFR
PRSS1
PRKCA
PRG2
PPBP
PPARG
POSTN
POMC
PLAU
PLA2G7
PLA2G4A
PLA2G2A
PLA2G1B
PK
PIK3CG
PHF11
PGR14
PGR
PF4
PDE5A
PDE4D
PDE4B
PDE4A
PDE4
PDE3A
PDE3
PDE11A
PDE
PDCD1LG2
PARP1
PAFAH
PAFAD
p38
OX40
ORMDL3
ORMDL2
ORMDL1
OPR
NR3C2
NR3C1
NR1I2
NR0B1
NPY
NPSR1-AS1
NPSR1
NPPA
NOS2
LTB4R
PLA2G1B
PTGIR
MAPK3
CYP2C19
CES1
PLA2G4A
CYP26A1
ALOX15
ESRRA
ADCY10
CYP2C9
C5AR1
ABCC1
ABCB1
PDE4A
PDE4B
CASP8
PRKCA
TGFB1
ADIPOQ
CAT
UGT1A1
CREB1
TIMP1
ABCG2
CCL2
CXCL10
CYP1B1
GSTM1
IL1A
IL1B
IRF1
PARP1
PTEN
SELE
SERPINE1
STAT1
VCAM1
CD14

GRIK5	NOS1
BBOX1	NOMO1
SLC6A12	NOD2
SLC22A6	NOD1
SLC7A5	NLRP3
KDM4E	NKAIN2
TACR1	NGF
KDM4C	NFKBIA
CPB2	NFKB2
ANPEP	NFKB1
ENPEP	NFKB
KDM1A	nAChR
CPA3	MYLK
TAAR1	MYL3
SLC15A1	MycB mmpL3
CPB1	MUC7
HRH3	MUC5B
HRH4	MUC5AC
GFPT1	MTTL1
HRH2	MS4A2
HRH1	MRT51
ODC1	MPO
BRD4	MPIP1
BRD2	MOP
BRDT	MMP9
BRD3	MMP2
CHIA	MMP-12
ACE	MMP12
REN	MMP1
F2	MME
CCNE1	MIR155
SHBG	MIR152
THRA	MIR148B
THRB	MIR148A
PTPRA	MIR126
NR1H4	MIF
APEX1	MCL1
MAPKAPK2	MC4R
GRB2	MBL2
KIF11	MARCKS
HDAC3	MAPK3
HCAR2	MAP3K9
DDO	M1 IgE
SIRT3	LY96
SIRT2	LTR
CAPN1	LTC4S
CASP1	LTB4R2
TGM2	LTB4R
ITGB1	LTA4H
ITGAV	LTA
CTSK	LT synth
AKR1A1	LGALS3
PTGDR2	LEP
KDM6B	L-CaC
PPARA	KNG1
CCKAR	KITLG
RHOA	KCNMB2
CNR2	KCNMB1

PSMB2	KCNMA1
PYGL	KC
PIK3CD	JUN
PIK3CB	JAK-3
CTSA	JAK-2
FFAR1	JAK2
EDNRB	JAK-1
CPA1	JAG2
ACE2	JAG1
GCK	ITK
TTR	ITGB6
NTSR1	ITGB2
PIK3CA	ITGAM
MKNK2	ITGAL
CREBBP	ITGA5
NR4A1	ITGA4/B1
ABL1	ITGA4
HSPA1A	IsoM
ITGAL	IRF4
CSNK2A1	IRF1
ITGA2B	IRAKM
FLT1	IRAK3
CDK5R1	IL9R
PDGFRB	IL9
MTNR1A	IL7
MAPK8	IL6R
ADAMTS5	IL6
KDR	IL5RA
FGFR1	IL5
ALOX5	IL4R
CA7	IL4
CA4	IL33
PPARD	IL31
FABP4	IL3
CPT1B	IL2RA
FABP3	IL25
MMEL1	IL23
FABP5	IL2
FNTA	IL1RN
MKNK1	IL1RL1
AGTR2	IL1R1
NTRK1	IL1B
ITGB7	IL1A
PAM	IL18R1
FDFT1	IL18
TDO2	IL17R
NEK2	IL17F
FPR2	IL17A
IDO1	IL17
AURKA	IL16
MMP13	IL13RA2
MMP14	IL13RA1
MMP7	IL13
AKR1C3	IL12RB2
DHODH	IL12RB1
MMP12	IL12B
KDM4D	IL11RA
IKBKB	IL11

P2RX7	IL10
NAAA	IL-1 synth/rele
PRKDC	IGHE
PDE9A	IGES
CDC25A	IgE
CDC25B	IFNG
ITGA2	ICOSLG
TBXAS1	ICOS
ADH1A	ICAM1
ADH1B	HSP90AA1
SRD5A1	HSP90
SRD5A2	HSP20
HSD17B7	HSD11B2
DPP7	HSD11B1
EPHX2	HRH1
FAAH	HR
DPP8	HPS1
DPP9	HNMT
CHRNA4	HM13
HTR2B	HLA-G
HTR7	HLA-DRB1
HTR1D	HLA-DRA
ADRB1	HLA-DQB1
HTR1E	HLA-DQA2
HTR6	HLA-DQA1
MMP3	HLA-DPB1
MPO	HLA-DPA1
CTSL	HLA-DOA
MME	HIST1H3A
ERAP2	His/p-LT rele
EPHA2	His rele
EPHB2	HDAC3
EPHA5	HDAC2
EPHA4	HAVCR2
EPHA8	HAVCR1
EPHA6	H4R
EPHA7	H1R
EPHB3	GSTP1
EPHA3	GSTM1
EPHB1	GSR
EPHB4	GSDMB
EPHA1	GS
EPHB6	GR
CTSC	GPRA
MC1R	GPR154
MIF	GP39
YARS	GLCCI1
XIAP	GIVA cPLA2
HLA-A	Gene
OPRK1	GATA3
LAP3	GAD2
FPR1	GAD1
CBX7	GABRB2
CBX4	GABRA2
SRC	G6PD
GALR2	FPR1
PDYN	FOXP3
HMGCR	FLG

PPIA	FLAP
HDAC1	FKBP5
NTSR2	FGFBP2
RRM1	FFAR2
PGGT1B	FCGR2B
TACR2	FCGR2A
HLA-DRB1	FCERG
CTSE	FCER2
BACE1	FCER1G
FAP	FCER1A
NPPA	FCAR
SIGMAR1	FASLG
CALCRL	FAS
PREP	FABP4
GPR142	F2RL1
AVPR1A	F2R
GHRL	F2
OXTR	ESRRA
PIN1	EPX
ITGA4	ELR accum
NPFFR1	ELANE
NPFFR2	EDN1
BACE2	DPP10
SPHK1	DENND1B
ACER2	DAP3
ASAH1	D2R
GHSR	CYSLTR2
BIRC3	CYSLTR1
BIRC2	CYP4A11
TRHR	CYP3A7
HDAC8	CYP3A5
MC4R	CYP3A43
MC3R	CYP3A4
STK3	CYP2E1
XPNPEP2	CYP2D6
CTSB	CYP2C9
QRFPR	CYP2C8
MLX	CYP2C19
TUBB1	CYP2B6
KLK1	CYP2A6
GALR1	CYP26A1
DRD3	CYP1B1
DRD4	CYP1A2
HSD11B1	CYP1A1
MTNR1B	CYP17A1
CTSS	CYP11B2
CTSV	CYP11B1
IMPDH2	CXCR4
EZH2	CXCR3
C3AR1	CXCR2
GABRB3	CXCR1
DUT	CXCL9
AHCY	CXCL8
P2RY1	CXCL5
UGCG	CXCL16
DYRK1A	CXCL12
CDK1	CXCL10
PDE10A	CXCL1

TACR3	CXADR
SCN9A	CX3CR1
PDE5A	CTSS
SHH	CTSL
PDE2A	CTSK
JAK2	CTSB
FLT3	CTLA4
GPR139	CST7
PSMB5	CSF3
GPBAR1	CSF2RB
TSPO	CSF2
PDE4D	CSF1
PIM2	CRHR1
SMO	CRH
CRHR1	CREB1
NOS1	CPNE1
CFD	CPN1
CELA1	CPA3
PDE7A	CP
GRIN2A	COX-2
SLC27A1	COX
PDGFRA	COMT
AURKB	COL26A1
PARP3	CMA1
TNKS	CLCA1
TLR4	CLC
LIMK2	CHRNA7
KCNJ5	CHRM5
KCNJ6	CHRM4
MAP3K8	CHRM3
TNKS2	CHRM2
GPR55	CHRM1
TRPA1	CHRM
CA1	CHR
METAP1	CHKB
CYP17A1	CHIT1
NR1I3	CHIA
TRPV1	CHI3L1
BCHE	CFTR
CNR1	CFB
HDAC6	CES1
ROCK2	CDSN
HDAC10	CDHR3
HDAC11	CD86
RAF1	CD80
BRAF	CD79A
NPY1R	CD74
LIPG	CD69
HSD17B3	CD44
PLAU	CD40LG
NEK1	CD40
YES1	CD4
MTOR	CD28
WEE1	CD274
CXCR3	CD209
SPHK2	CD14
RET	CCR8
CCR8	CCR7

ERN1	CCR6
DUSP3	CCR5
HSP90AA1	CCR4
CDC7	CCR3
SGK1	CCL7
MYLK	CCL5
LIMK1	CCL4
CDC42BPA	CCL3
TNK2	CCL26
DMPK	CCL24
PKN2	CCL22
TLR9	CCL2
MPEG1	CCL18
KAT2B	CCL17
F9	CCL13
KIT	CCL11
PFKFB3	CBX5
SCD	CBR1
STK17B	CAV3
MAP4K4	CAT
AKR1B10	CASP8
FABP2	CAMP
VDR	CALCA
PTGER2	CALC
UGT2B7	CA-IV
PHF8	C5AR1
SERPINA6	C5
G6PD	C3AR1
CYP19A1	C3
NPC1L1	BTK
KDM5C	BRCA2
KDM2A	BPIFA1
POLB	BMP6
PTGFR	BKbeta4
GSTK1	BHR1
HAO1	BGLAP
HSD11B2	BDNF
FFAR4	BDKRB2
CDC45	BCHE
PTGER4	ATP2A2
PTGER3	ATIII
TBXA2R	ASRT8
SLC22A12	ASRT7
CA12	ASRT6
PTPRC	ASRT5
RBP4	ASRT4
PTGDR	ASRT3
SLC16A1	ASRT2
RARA	ASRT1
RARG	AS1
RARB	ARG2
CMA1	ARG1
CTSG	AREG
CXCL8	AOC3
SAE1	ANXA1
STS	ANGPT2
BMP1	ANGPT1
FABP1	AMICA1

GSR
RXRB
RXRG
PSEN2
FOLH1
HDAC2
ALOX12
PTPN2
TERT
PTPN6
CES2
LTB4R
RORC
PTPRF
CD81
PRKCH
PTGER1
CYP51A1
PLA2G1B
PTGIR
ACP1
MAPK3
NR1H3
PTPN11
RORA
CYP2C19
CES1
CTNNB1
RORB
PLA2G4A
CYP26B1
CYP26A1
ALOX15
KEAP1
PTGES2
NR0B2
OXER1
AKR1C1
GLRA1
GCGR
PRKCD
ESRRA
ESRRB
GPER1
ADCY10
CYP2C9
NR1H2
C5AR1
KCNA3
SLC6A9
KCNA5
PER2
S1PR5
S1PR4
ABCC1
ABCB1
HNF4A
GLUL
ALRH
ALOX5AP
ALOX5 mRNA
ALOX5
ALOX15B
ALOX15
ALG9
ALB
AKR1D1
ADRB3
ADRB2
ADRB1
ADRA2C
ADRA2B
ADRA2A
ADRA1D
ADRA1B
ADRA1A
ADORA3
ADORA2B
ADORA2A
ADORA1 mRNA
ADORA1
ADIPOQ
ADH5
ADCY9
ADCY10
ADAM8
ADAM33
ADA
ACTG2
ACKR3
ACE
ABCG2
ABCC2
ABCC1
ABCB11
ABCB1
ABCA1
AAA1
5-LOX

ENPP2
DAGLA
EPHX1
TRPM8
PRKAG1
GCG
ABHD6
PDE4A
PDE4B
PSEN1
BAX
CASP8
MAP2
PON1
PRKCA
TGFB1
ADH1C
CTRB1
MAOA
MAOB
CDKN2
TOP2B
F10
AHR
APOD
CRK2
CYCS
FOSL1
FOSL2
HIF1A
IGF2
NFATC1
NOX5
TDRD7
ADIPOQ
APOB
BAD
CAT
GOT1
LDLR
MTTP
PLB1
SOAT1
SOAT2
SOD1
SREBF1
UGT1A1
CD163
CREB1
TIMP1
NCF1
OLR1
PYGM
RHO
ABCG2
ACACA
ACP3
CAV1

CCL2
CHEK2
CHUK
CLDN4
COL1A1
COL3A1
CRP
CTSD
CXCL10
CXCL11
CXCL2
CYP1B1
DCAF5
DIO1
DUOX2
E2F1
E2F2
EGF
EIF6
ELK1
ERBB3
F3
GJA1
GSTM1
GSTM2
HAS2
HK2
HSF1
HSPA5
HSPB1
IGFBP3
IL1A
IL1B
IRF1
MGAM
NFE2L2
NKX3-1
NPEPPS
NQO1
PARP1
PCOLCE
PLAT
POR
PRKCB
PSMD3
PTEN
RASA1
RASSF1
RUNX1T1
RUNX2
SELE
SERPINE1
SPP1
STAT1
SULT1E1
THBD
VCAM1
PPP3CA

SLPI
CD14
LBP
ATP5F1B
HSD3B1
HSD3B2
MT-ND6
PKIA
SIRT1
MAPK10
