# Supplementary material for: Network Pharmacology Combined with Molecular Docking and Experimental Verification Reveals the Bioactive Components and Potential Targets of Danlong Dingchuan Decoction against Asthma
Source: Evid Based Complement Alternat Med. 2022 Feb 10;2022:7895271. doi: 10.1155/2022/7895271 (PMC8853800; doi:10.1155/2022/7895271)
Supplement: Supplementary Materials — Table S1: basic information on active compounds in Danlong Dingchuan Decoction. Table S2: gene targets of Danlong Dingchuan Decoction and asthma. Figure S1: graphical abstract of the paper. [file supplementary-graphical abstract.pdf › 7895271.f1/supplementary-graphical abstract.pdf]

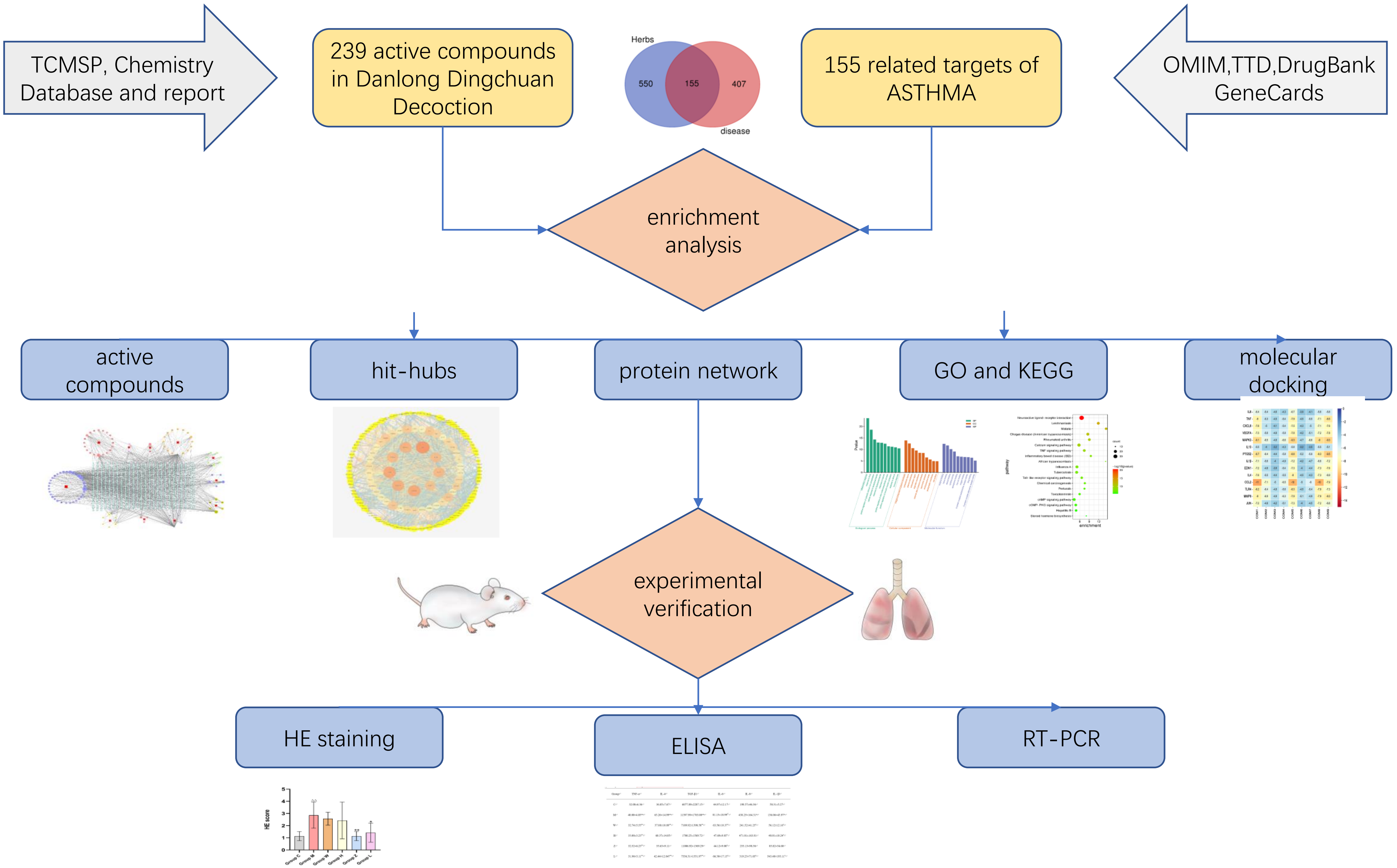
TCMSP, Chemistry Database and report
239 active compounds in Danlong Dingchuan Decoction
Herbs
550
155
407
disease
155 related targets of ASTHMA
OMIM,TTD,DrugBank GeneCards
enrichment analysis
active compounds
hit-hubs
protein network
GO and KEGG
molecular docking
experimental verification
HE staining
ELISA
RT-PCR
HE score
